# The Forkhead Transcription Factor Foxi1 Is a Master Regulator of Vacuolar H^+^-ATPase Proton Pump Subunits in the Inner Ear, Kidney and Epididymis

**DOI:** 10.1371/journal.pone.0004471

**Published:** 2009-02-13

**Authors:** Hilmar Vidarsson, Rickard Westergren, Mikael Heglind, Sandra Rodrigo Blomqvist, Sylvie Breton, Sven Enerbäck

**Affiliations:** 1 Center of Medical Genetics, Institute of Biomedicine, The Sahlgrenska Academy, Göteborg University, Göteborg, Sweden; 2 Center for Systems Biology, Program in Membrane Biology/Nephrology Division, Massachusetts General Hospital, Harvard Medical School, Boston, Massachusetts, United States of America; Katholieke Universiteit Leuven, Belgium

## Abstract

The vacuolar H^+^-ATPase dependent transport of protons across cytoplasmic membranes in FORE (**fo**rkhead **re**lated) cells of endolymphatic epithelium in the inner ear, intercalated cells of collecting ducts in the kidney and in narrow and clear cells of epididymis require expression of several subunits that assemble into a functional multimeric proton pump. We demonstrate that expression of four such subunits A1, B1, E2 and a4 all co-localize with the forkhead transcription factor Foxi1 in a subset of epithelial cells at these three locations. In cells, of such epithelia, that lack Foxi1 we fail to identify any expression of A1, B1, E2 and a4 demonstrating an important role for the transcription factor Foxi1 in regulating subunit availability. Promoter reporter experiments, electrophoretic mobility shift assays (EMSA) and site directed mutagenesis demonstrate that a Foxi1 expression vector can *trans*-activate an a4-promoter reporter construct in a dose dependent manner. Furthermore, we demonstrate using chromatin immunoprecipitation (ChIP) assays that Foxi1-dependent activation to a large extent depends on *cis*-elements at position −561/−547 in the a4 promoter. Thus, we provide evidence that Foxi1 is necessary for expression of at least four subunits in three different epithelia and most likely is a major determinant for proper assembly of a functional vacuolar H^+^-ATPase complex at these locations.

## Introduction

Vacuolar-type H^+^-ATPases (v-ATPases) consist of two major subunits: a membrane anchoring V_0_ domain and a catalytic V_1_ domain. These multimeric enzymes are mediators of both intra- and extracellular vectorial proton transport in all eukaryotic cells. This type of proton pump is also enriched for in the plasma membrane of cells specialized in proton secretion such as FORE cells of the endolymphatic sac and duct [1], [2], intercalated cells of the collecting duct in the kidney [3] and narrow and clear cells of epididymis [4]. While there is a generic structure of v-ATPase proton pumps derived from assemblage of ubiquitously expressed subunits e.g. A1 and E2 many cell types present a particular holoenzyme composition based on tissue or cell specific expression of subunit isoforms e.g. B1 and a4 that are expressed in epithelia of inner ear, kidney and epidiymis [5]. The V_0_ domain is involved in proton translocation and consists of subunits designated by lower case letters such as a4. A large cytosolic complex is formed by the V_1_ domain and here subunits are labeled by capital letters A–H. Subunits A1 and B1 interact with ATP while E2 is part of a stalk-like structure that connects the V_0_ and V_1_ domains [6]. Mutations in genes encoding several of these “tissue specific” subunits have been shown to cause human disease such as recessive distal renal tubular acidosis with (*Atp6v1b1*, encodes subunit B1; [7]) or in most cases without deafness (*Atp6v0a4*, encodes subunit a4; [8]). In spite of a growing body of information regarding structure – function relationships of v-ATPase [9], intracellular targeting and recycling [10] as well as how the coupling ratio between ATP hydrolysis and proton transport is modulated [11] little is known regarding upstream regulators of subunit gene expression.

This is particularly interesting since some of the subunits are ubiquitously expressed whereas others display a more restricted expression profile. Are ubiquitously expressed subunits regulated by a set of common transcription factors in a generic way similar in all tissues of expression or is the regulation of these subunits to some extent depending on distinct sets of transcriptional regulators varying between different tissues/celltypes? In an effort to address this question we studied the role of a tissue specific transcription factor, Foxi1, in regulation of both specifically e.g. B1 and a4 as well as ubiquitously expressed subunits e.g. A1 and E2.

We can demonstrate an important role of Foxi1 as regulator of B1 subunit expression in FORE cells of the endolymphatic epithelium. Furthermore, we demonstrate that Foxi1 is necessary for expression of the v-ATPase subunits A1, E2 and a4 in endolymphatic duct, kidney collecting duct and epididymal epithelia. Several potential Foxi1-binding *cis*-elements have been identified in the human a4 promoter. A cluster of three such sites is shown to interact with Foxi1, which through this interaction trans-activates an a4 promoter reporter construct. Mutations that inhibit interactions also significantly reduce Foxi1 dependent reporter gene activation. Moreover, mRNA in situ experiments demonstrate loss of mRNA encoding the ubiquitously expressed subunits A1 and E2 in kidney epithelia that lacks Foxi1. The data presented here establishes Foxi1 as a potential genetic regulator of subunit availability for at least four v-ATPase subunits all expressed in endolymphatic duct, kidney collecting duct and epididymal epithelia.

## Results

### Foxi1 is required for and co-localizes with expression of A1, B1, E2 and a4 subunits in endolymphatic sac and duct epithelia

**Figure 1:**
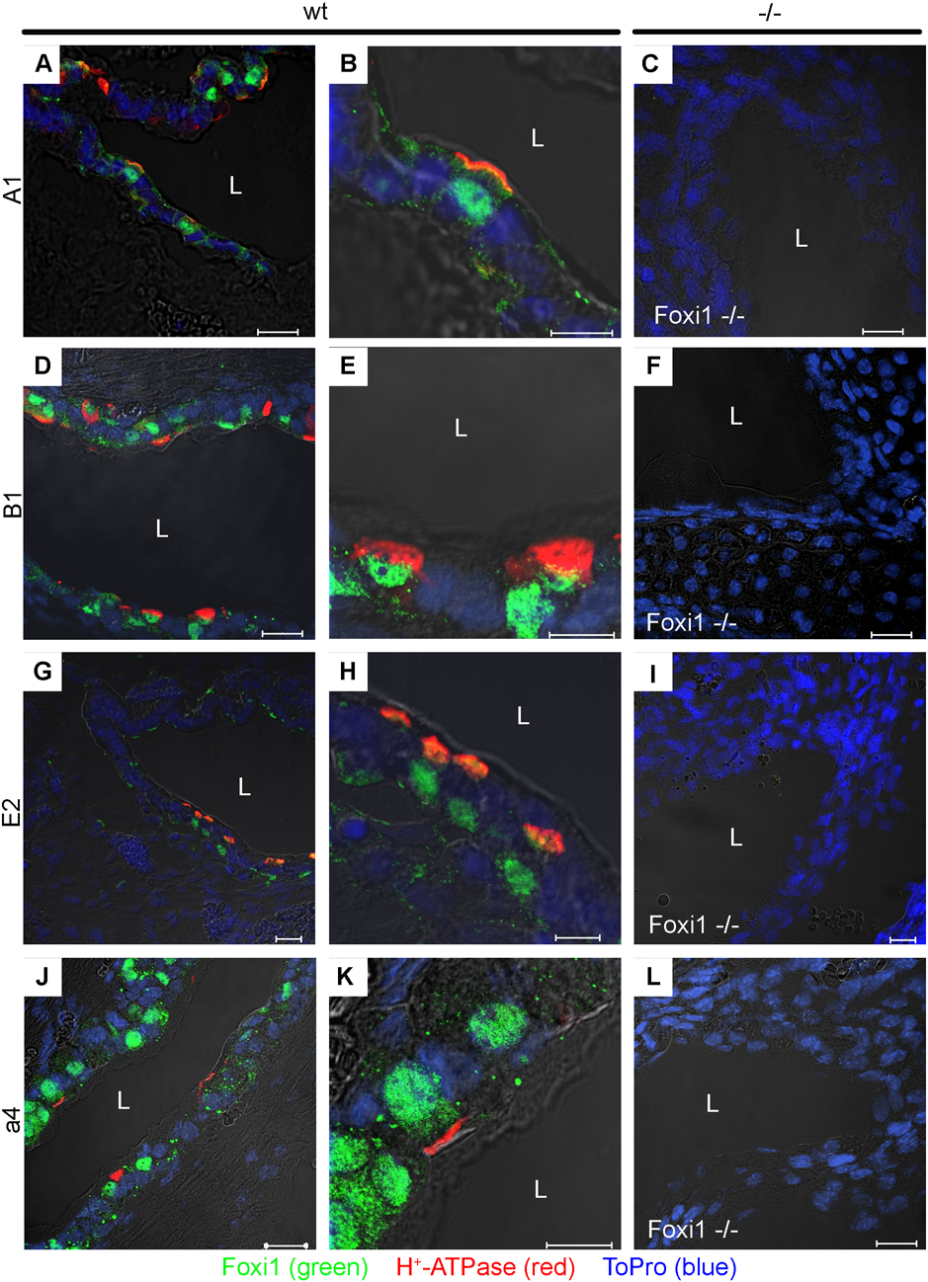
Confocal analysis of Foxi1 and H^+^-ATPase subunits A1, B1, E2 and a4 expression in wt and Foxi1−/− endolymphatic sac (ES) epithelium. Confocal images of inner ear sections from wt and Foxi1−/− mouse embryos (E16.5). Fluorescence images of wt ES tissue sections stained with a specific antibody against Foxi1 (green) and the H^+^-ATPase subunit A1 (red; A–B), subunit B1 (red; D–E), subunit E2 (red; G–H) and subunit a4 (red; J–K). The nuclei (blue) were visualized using To-Pro 3. Merged images reveal that cells with a strong staining of each of the H^+^-ATPase subunits also are positive for nuclear Foxi1 staining. In sections from Foxi1−/− ES no staining could be identified for A1 (C), B1 (F), E2 (I) or a4 (L). Scale bars 20 µm (A, D, G, J, C, F, I and L) and 10 µm (B, E, H and K) (L: lumen).

As can be depicted from figure 1 both the “tissue specific” subunits B1 and a4, expressed in the inner ear, kidney and epididymis, as well as the ubiquitously expressed isoforms A1 and E2 co-localizes with Foxi1 specific immunostaining. While the different subunits as expected are expressed at the apical aspect of epithelial FORE cells Foxi1 staining is localized to the nuclei (Fig. 1 B, E, H, K). Epithelia that lack Foxi1 expression are completely void of B1, a4 as well as A1 and E2 expression (Fig. 1 C, F, I, L). This strongly suggests that Foxi1 is a prerequisite for expression of these subunits. It also agrees well with previous findings indicating that Foxi1 mRNA is expressed in a subset of epithelial cells in the endolymphatic duct and sac called FORE cells [1].

### Foxi1 co-localizes with A1, E2 and a4 subunits in intercalated cells of the kidney and is required for expression

**Figure 2 pone-0004471-g002:**
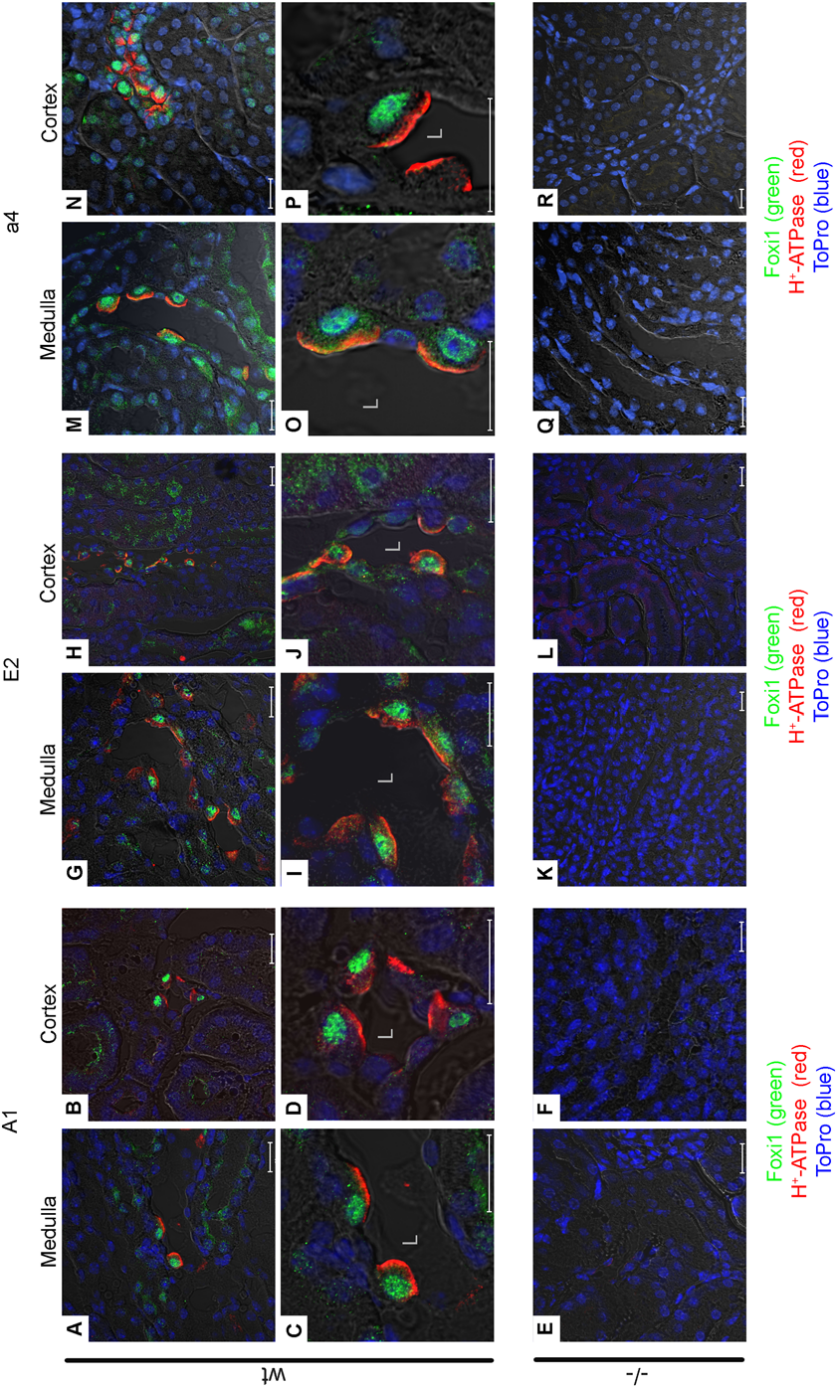
Confocal analysis of Foxi1 and the H^+^-ATPase subunits A1, E2 and a4 expression in wt and Foxi1−/− distal nephrons. Confocal images of wt and Foxi1−/− kidney sections. Double staining with Foxi1 and anti H^+^-ATPase antibodies showed exclusive expression of Foxi1 protein (green) in cells of distal nephron cells co-expressing subunits A1 (red; A–D), E2 (red; G–J) and a4 (red; M–P), in both cortex as well as medulla. Foxi1 is localized to the nuclei, which are stained blue (TOPRO 3), while the ATPase subunits are found apically, a4 (O–P) or both apically and in the cytosol, A1 (C–D) and E2 (I–J). No expression of ATPase subunits were detected in epithelial cells from Foxi1−/− kidneys, neither in medulla (E, K, Q) nor cortex (F, L, R). Scale bars 20 µm (L: lumen).

The subunits A1, E2 and a4 are expressed in a subset of epithelial kidney cells (Fig. 2). Based on previous findings in which Foxi1 has been established as a marker for intercalated cells [12] we can based on co-localization with Foxi1 determine that A1, E2 and a4 all are expressed in intercalated cells. In sections from both medulla and cortex we demonstrate a complete co-localization between Foxi1 and the subunits A1, E2 and a4. These subunits display a luminal staining pattern while the Foxi1 signals reside in the nuclei (Fig. 2 A–D, G–J, M–P). Kidneys from mice lacking Foxi1 have no detectable A1, E2 or a4 (Fig. 2 E, F, K, L, Q, R). The overlap in expression between these subunits and Foxi1 appears to be complete i.e. all Foxi1 cells studied are positive for A1, E2 and a4. It is known from previous work that the B1 subunit is expressed in intercalated cells and that its expression requires Foxi1 [12].

### Foxi1 co-localizes with and is required for expression of A1, E2 and a4 subunits in narrow and clear cells of epididymis

**Figure 3:**
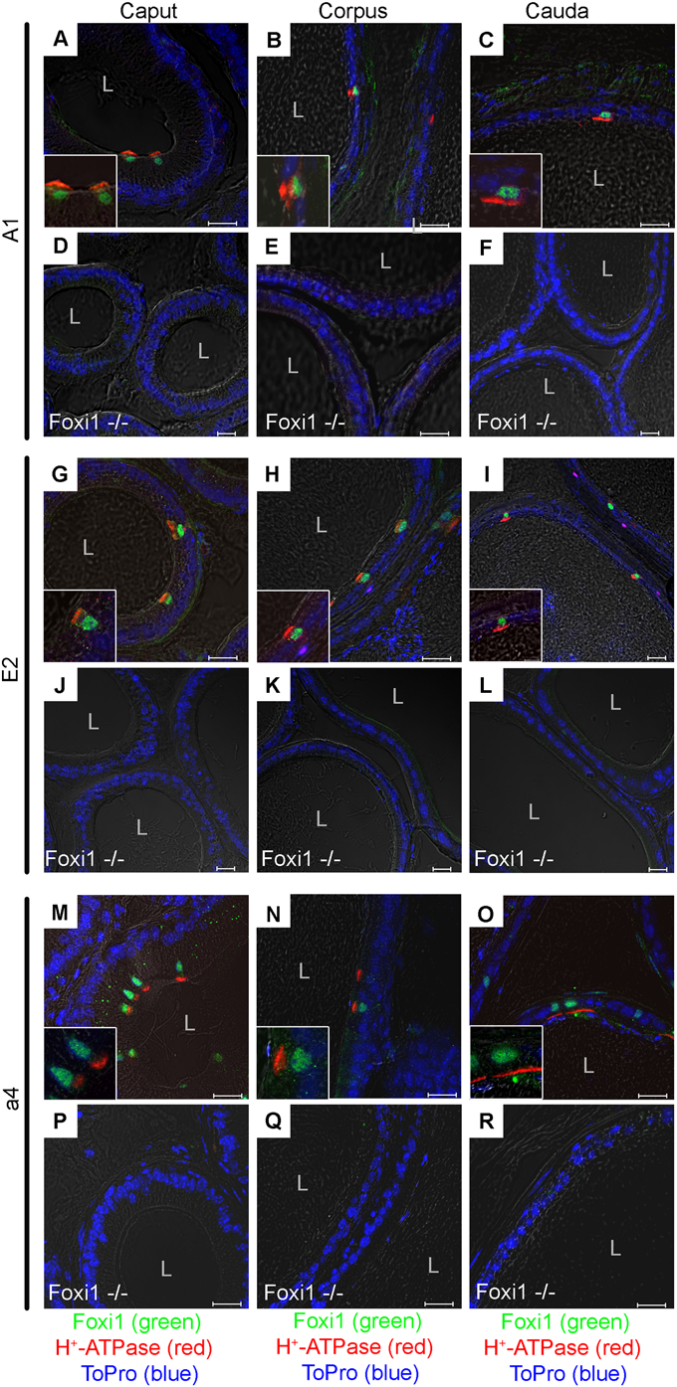
Confocal analysis of Foxi1 and H^+^-ATPase subunits A1, E2 and a4 expression in wt and Foxi1−/− epididymides. Confocal images of wt and Foxi1 −/− epididymal sections. In confocal images of wt epididymal sections, all three ATPase subunits (red) were exclusively found in Foxi1 (green) immunoreactive epididymal cells of caput (A, G, M), corpus (B, H, N) and cauda (C, I, O). Foxi1 (green) is localized to the nuclei (blue, TOPRO3), while the ATPase subunits are found on the apical/luminal (L) side of the cells. Staining of Foxi1−/− tissue sections showed no detectable A1 (D, E, F), E2 (J, K, L) nor a4 (P, Q, R) subunit expression in Foxi1 deficient epididymal tissue sections. Scale bars 20 µm (L: lumen).

Here we demonstrate that the subunits A1, E2 and a4 are expressed in epididymis and that there is a clear overlap in expression between Foxi1 and these subunits (Fig. 3). Since Foxi1 is a marker for proton secreting narrow and clear cells we can conclude that not only the B1 subunit, as has previously been shown [13], but also A1, E2 and a4 are expressed in these cell types. Staining patterns of A1, E2 and a4 are similar to what has been reported for the B1 subunit [13]. Epididymes lacking Foxi1 do not express any of the investigated subunits, a result that extends the previous findings of Foxi1 as a master regulator of subunit expression in endolymphatic epithelium and distal tubuli/collecting ducts of the kidney to also include the epididymal epithelium.

### Foxi1 interacts with and *trans*-activates an *ATP6V0A4* promoter reporter construct *in vitro*

**Figure 4:**
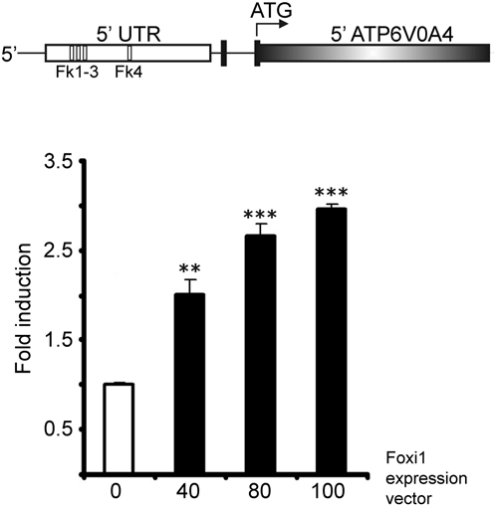
Foxi1 can activate an *ATP6V0A4* promoter reporter construct. Sequence analysis of the *ATP6V0A4* upstream region reveals four putative FOXI1 sites located at −561/−547 (Fk1-3) and −358/−352 (Fk4) relative to start of transcription (NCBI: NC_000007.12, NM_130841). A −754 ATP6V0A4 promoter construct was transfected into COS7 cells using increasing amounts of Foxi1 expression plasmid. A significant step-wise induction is shown.

**Figure 5:**
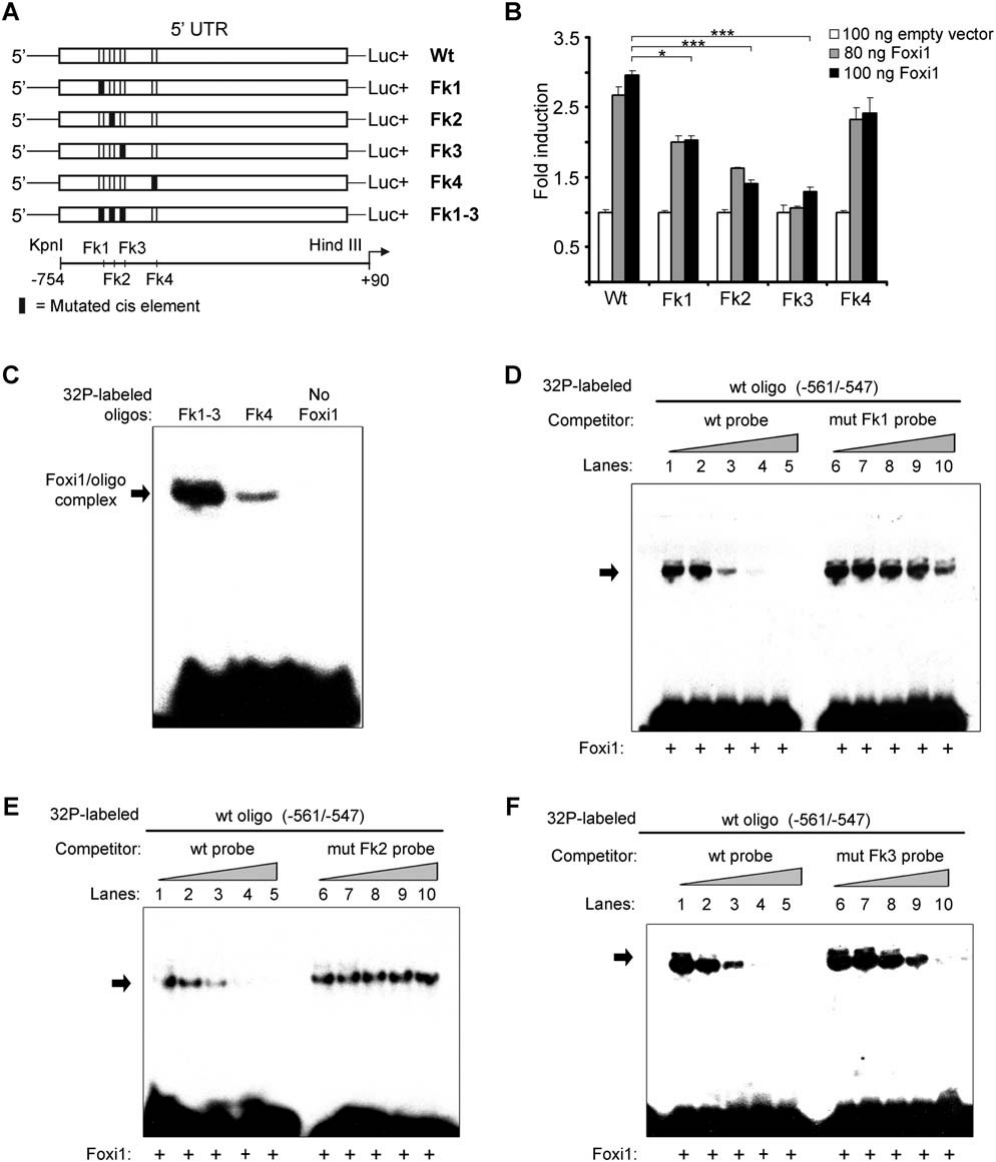
Foxi1 interact and activate *ATP6V0A4* promoter reporter construct at putative forkhead binding sites Fk1-3. (A) Core TTT-triplets of potential forkhead sites were mutated to GGG according to schematic picture. (B) Mutations reduce Foxi1-depedent reporter activation. While Fk3 mutations seem to abolish this activation the effects on Fk1-2 are intermediate and Fk4 seems to contribute very little to Foxi1-dependent reporter gene activation. (C) EMSA using *in vitro* transcribed/translated Foxi1 demonstrates interactions with [^32^P] labeled oligonucleotides harboring the −561/−547 (Fk1-3) and −358/−352 (Fk4) regions. The comparatively weak interaction demonstrated for Fk4 correlates well with the modest effects on reporter gene activation seen in Fig. 5 B. (D–F) EMSA showing the −561/−547 interaction is competed for with either wt or mutated oligonucleotides specific for the Fk1, 2 and 3 interactions. As can be deduced, there is a higher sensitivity in EMSA competition experiments using wt probes as compared with mutated probes – typical of a sequence specific significant interaction.

**Figure 6:**
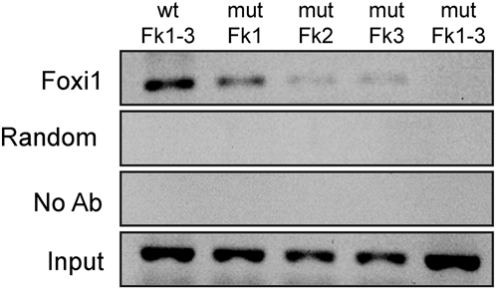
Chromatin Immuoprecipitation (ChIP) analysis. Chromatin immunoprecipitation confirm presence of Foxi1- ATP6V0A4 promoter interaction at FK1-3. Transfected 3T3-L1 cells were fixed, and chromatin was prepared by sonication. After preclearance with Protein G Sepharose the chromatin was immunoprecipitated with or without an Anti-6X His tag antibody (Foxi1, No Ab). The purified precipitated DNA was used as template for PCR reactions with specific primers covering sequence with Foxi1 binding sites (Fk1-3) or unspecific primers (Random). ChIP analysis reveal a strong interaction of the ATP6V0A4 promoter with wt Foxi1 (Lane 1). The interaction is weakened using promoter with mutated FK1 (Lane2) and even more weakened with Fk2 and 3 (Lanes 3–4). Using the triple mutated promoter Fk1-3 (Lane 5) no interaction is detected.

**Figure 7:**
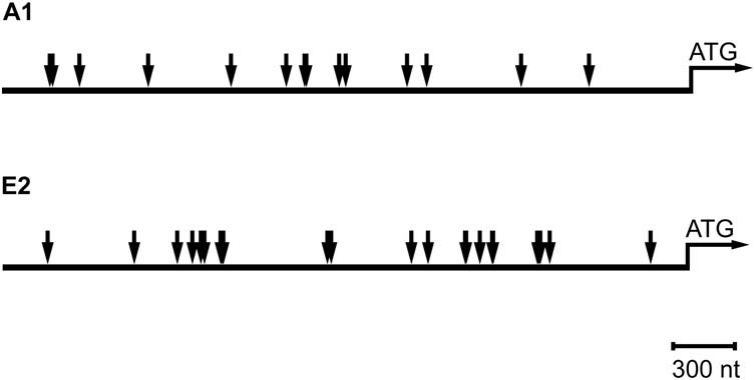
In silico identification of forkhead sites in genes encoding subunits A1 and E2. Representation of potential forkhead binding sites in the upstream regions of genes encoding the A1 and E2 subunit. In the depicted 3 kb region 14 and 25 sites where identified for A1 and E2, respectively.

To investigate if Foxi1 regulates expression of the a4 subunit we studied the a4 promoter sequence and identified a cluster of three potential forkhead binding *cis*-elements (Fk1-3) adhering to the forkhead binding consensus sequence (Fig. 4; T(g/a)TTT(g/a) (t/c); [14]). Reporter constructs encompassing these sites show a dose-dependent Foxi1 activation (Fig. 4). Some two hundred bases downstream from this cluster we found another potential forkhead binding site (Fk4; Fig. 4). When any of the three sites in the cluster were mutated there was a significant drop in reporter gene activity, the most prominent effect was noted for the Fk3 site (Fig. 5A, B). While mutations at this site essentially blocked Foxi1 dependent inducibility the Fk2 site reduced Foxi1 ability to *trans*-activate by approximately half and finally Fk3 reduced this effect by about a third (Fig. 5A, B). Mutations at Fk4 did not significantly affect reporter activity. In EMSA experiments we could demonstrate a distinct mobility shift using a labeled double stranded oligonucleotide harboring the Fk1-3 cluster (Fig. 5C). We could also observe a much weaker interaction for Fk4 (Fig. 5C) that agrees well with the effects these sites have on total Foxi1 mediated reporter gene activation (Fig. 5B). To investigate the nature of these interactions we performed a series of EMSA experiments using the same mutations as in the transfection experiments and wt (wild type) sequences (Fig. 5D–F). While it can be deduced from figures 5D, E and F that the unlabeled wt probe effectively competes for the Fk1, 2 and 3 protein interactions at concentrations of approximately 10-fold molar excess, compatible with *bona fide* interactions, the mutated Fk1, 2 and 3 probes are significantly less efficient as competitors and display significant competition only at ∼100-fold molar excess in most instances, a finding that agrees well with transfection data (Fig. 5B). Taken together, these results suggest that Foxi1 is able to interact with the *ATP6V0A4* promoter at position −561/−547 in a sequence specific manner and this argues in favor for direct Foxi1-mediated regulation of *ATP6V0A4* gene expression in kidney, inner ear and epididymis. We have previously shown in transfection experiments that FOXI1 interacts with a key *cis*-element in the *ATP6V1B1* promoter, at −102/−96, and that this interaction is required for a dose-dependent activation by FOXI1 [13]. In conclusion, this is compatible with a direct role for Foxi1 in transcriptional regulation of a4 and B1 subunits and establishes these genes as Foxi1 downstream targets. In chromatin immunoprecipitation experiments, aimed to study *in vivo* interactions at the Fk1-3 cluster, we confirm that Foxi1 interactions are strongest at Fk3, slightly weaker at Fk2 and weakest at Fk1 (Fig. 6). When all three sites in the Fk1-3 cluster are mutated we cannot detect any Foxi1 interaction (Fig. 6). An *in silico* analysis of upstream regions in the A1 and E2 genes demonstrates the presence of several potential forkhead binding sites conforming to the consensus sequence t(g/a)TTT(g/a)(t/c) (Overdier et al., 1997; Fig. 7).

### Foxi1 is necessary for A1 and E2 encoding mRNA expression in intercalated cells of the kidney depend

**Figure 8:**
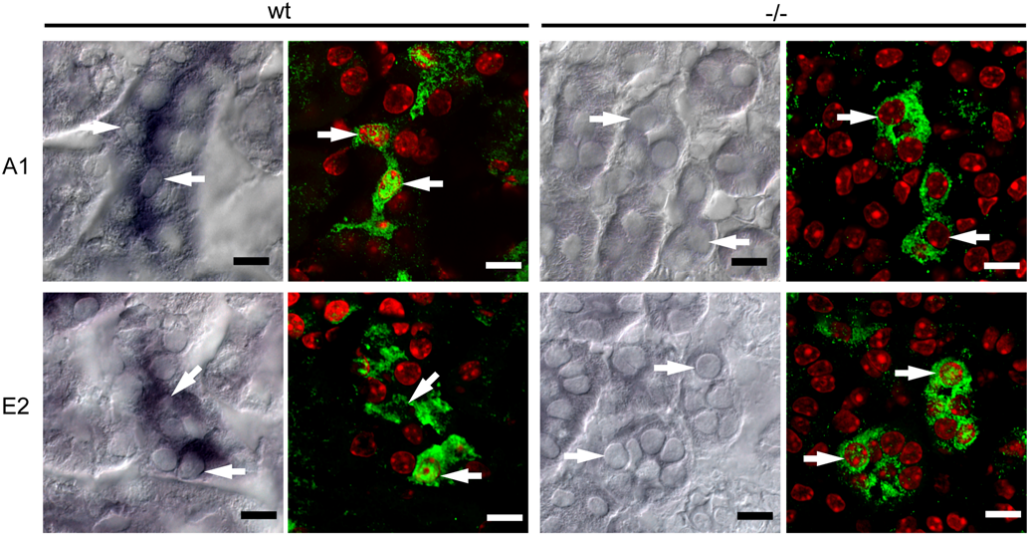
mRNA in situ hybridization and immunohistochemistry on kidney sections from wt and Foxi1−/− mice. Combined *in situ* hybridization and immunofluorescence. DIG labeled cRNA probes for A1 and E2 were hybridized to wt and Foxi1−/− kidney sections and the same sections were then subjected to immunofluorescent staining with an anti-CAII specific antibody (green) and the nuclear marker Topro3 (red). CAII expressing cells in wt kidney displayed positive hybridization signal for both A1 and E2 probes while in Foxi1−/− kidney sections, no hybridization signal could be detected. White arrows point to the same CAII positive cells in images from in situ hybridization and immunofluorescence. Scale bars 10 µm.

While overlapping expression patterns of the transcription factor Foxi1 and the a4 and B1 subunits to some degree is suggestive of a regulative role for Foxi1 it is somewhat surprising that the ubiquitously expressed subunits A1 and E2 also appear to be dependent on Foxi1 expression (Fig. 1–3). Although proper assembly has been shown to be important for both intracellular targeting/localization and stability of subunits [15], [16], [17] specific regulation of ubiquitously expressed genes/proteins constitutes another possible mode of regulation. To address the general question: whether this is due to an Foxi1 mediated effect on mRNA levels or if lack of the tissue specific subunits B1 and a4 would lead to destabilization of the multimeric complex and as a consequence hereof enhanced degradation of its constituents. We performed mRNA in situ hybridization on kidney sections from wt and Foxi1 −/− mice using mRNA anti-sense probes for A1 and E2. On the same sections, as described previously [12] we performed IHC using an antibody directed against carbonic anhydrase II (CAII) a marker for intercalated cells in the kidney (Fig. 8). While CAII positive intercalated cells are present in both wt and Foxi1−/− kidneys A1 and E2 mRNA can only be detected in wt kidneys. Thus, even though A1 and E2 belong to a class of more broadly expressed subunits they appear to require Foxi1 for expression of their mRNA in these cell types.

## Discussion

To acidify intracellular organelles all eukaryotic cells rely on expression of multimeric vacuolar H^+^-ATPase proton pumps. In some specialized cell types v-ATPase proton pumps are present in the outer cellular membrane to support vectorial proton secretion over the apical plasma membrane. While the former function is vital for cellular adaptation and ultimately survival the latter supports specialized functions of the multi-cellular organism. Examples hereof include: *(i)* intercalated cells of the kidney distal tubuli and collecting ducts where proton transport is essential for maintaining proper systemic acid/base homeostasis loss of this function leads to distal renal tubular acidosis (dRTA; [7], [8]), *(ii)* FORE cells in the endolymphatic epithelium of the inner ear secretes protons into endolymph a process important for maintaining appropriate ionic composition which in turn is vital for conversion of acoustic sound waves into neuronal action potentials a process critical for hearing [1], [7], [8] *(iii)* narrow and clear cells of epididymis secretes protons into the epididymal lumen generating a low pH that is crucial for post-testicular maturation of spermatozoa which in turn is necessary for male fertility [13]. At these three locations the generic pattern of ubiquitously expressed subunits are complemented by expression of cell specific subunits such as a4 and B1. The importance of these specialized functions is underscored by the fact that mutations in the gene encoding the B1 subunit, *Atp6v1b1*, are associated with childhood sensorineural hearing loss and dRTA. This is also true for the gene encoding the a4 subunit, *Atp6v0a4*, although deafness is less common and appears to have a later onset, usually in the third to fourth decade [18].

The a4 subunit is expressed in Foxi1 positive cells of endolymphatic epithelium, intercalated cells in the kidney and narrow and clear cells of epididymis (Fig. 1–3). The expression appears to be restricted to Foxi1 positive cells and located in the apical aspect of such cells as expected for a factor involved in proton transport over the luminal plasma membrane (Fig. 1–3). It is also clear that in epithelia lacking Foxi1 there is no detectable expression of the a4 subunit (Fig. 1–3). To further investigate the role of Foxi1 in regulation of a4 subunit expression we identified four potential Foxi1 *cis*-elements in the Atp6v0a4 promoter Fkh1-4 (Fig. 4). While Fk4, if anything, only marginally contributes to reporter gene activity the Fk1-3 cluster seems to be the major site for Foxi1 dependent regulation of a4 transcription (Fig. 5). Site directed mutagenesis of Fk3 makes the a4 promoter virtually insensitive to Foxi1 expression in contrast to the very limited effects on Foxi1 inducibility noted for mutations of Fk4 (Fig. 5A). EMSA experiments demonstrate significant DNA-protein interaction with Fk1-3 and only limited interactions with Fk4 (Fig. 5C) supporting the conclusion based on transfection data that the former site is predominant in terms of Foxi1-mediated regulation of a4. Furthermore, EMSA analysis of the Fk1-3 complex shows a pattern of significantly reduced ability of the mutated oligonucleotides to compete for wt interactions (Fig. 5 D–F) a finding that supports the transfection data and argues in favor of these sites as biologically significant Foxi1 targets. Chromatin immunoprecipitation experiments (Fig. 6) also delineate the Fk1-3 complex as a site for Foxi1 interactions *in vivo*. In conclusion, these results support a direct role of Foxi1 in regulation of the a4 subunit in FORE cells of the inner ear endolymphatic epithelium, intercalated cells of the kidney and narrow and clear cells in epidiymis. The presence of 14 and 25 potential forkhead binding sites in the upstream regions of the genes encoding the A1 and E2 subunits suggests the possibility of Foxi1 as a potential regulator of these subunits (Fig. 7). A fact supported by the finding that kidney epithelium that lacks Foxi1 while retaining expression of carbonic anhydrase II, a marker for intercalated cells, have no detectable levels of neither A1 nor E2 mRNA (Fig. 8).

**Figure 9:**
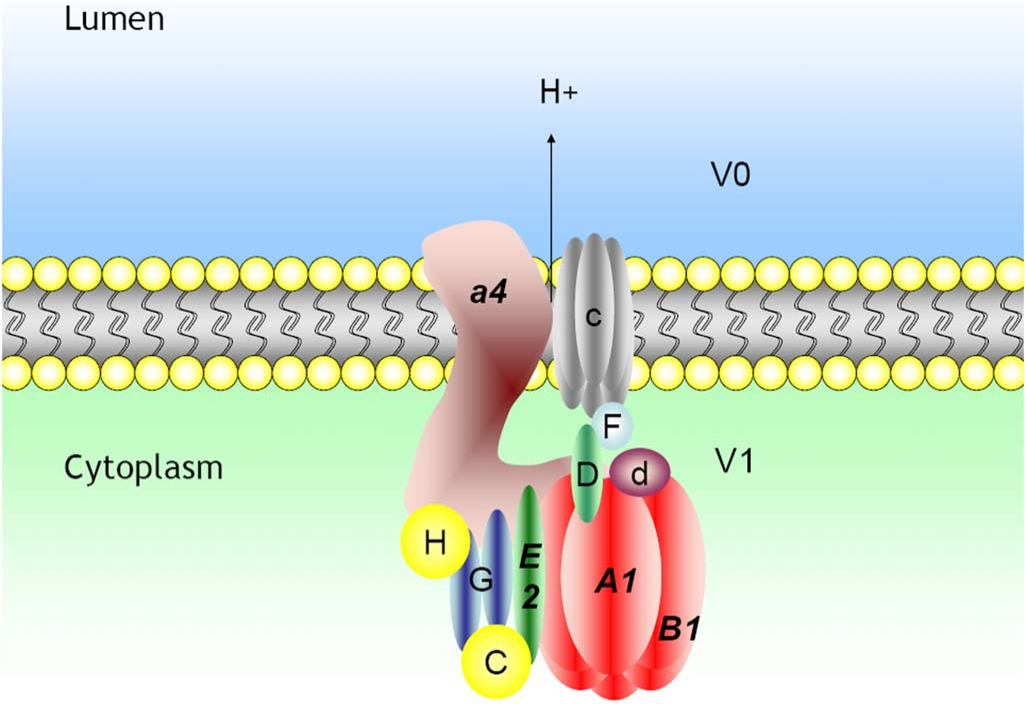
Schematic illustration of the vacuolar H^+^-ATPase proton pump. Subunits specifically expressed in endolymphatic epithelium of the inner ear, intercalated cells of kidney and narrow and clear cells of the epididymis in bold.

We have previously shown that Foxi1 through direct DNA-protein interactions, appears to be crucial for transcription of the Atp6v1b1 gene [13]. Mutations in a forkhead *cis*-element, at −102/−96, in the B1 promoter abolish both DNA-protein interactions and Foxi1-dependent induction of promoter reporter gene activity. This finding is supported by the absence of B1 protein in intercalated cells of the kidney and narrow and clear cells of epididymal epithelium in mice that lack Foxi1 [12], [13]. In this study we extend this observation to also include the endolymphatic epithelium of the inner ear (Fig. 1 D, E). Based on the following observations we conclude that Foxi1 is necessary for expression of the B1 subunit in these three epithelia: *(i)* B1 and Foxi1 co-localize in these epithelia ([12], [13]; Fig. 1 D, E), *(ii)* Foxi1 interacts with and can *trans*-activate the B1 promoter [13], *(iii)* in epithelia lacking Foxi1 there is no B1 expression ([12], [13]; Fig. 1 D, E). We also present data indicating that Foxi1 is needed for transcription of the ubiquitously expressed subunits A1 and E2 in the kidney (Fig. 8). The structure of the multimeric vATPase proton pump is outlined in figure 9.

Data presented here strengthens the role of Foxi1 as an important regulator v-ATPase function at three very specific locations: FORE cells in the endolympahtic epithelium, intercalated cell in the kidney and narrow and clear cells of epididymis. Several familial and sporadic cases of deafness and dRTA have been linked to mutations in B1 and a4 subunits [7], [8], [18]. In a recent publication the first human data on mutations in FOXI1 was presented demonstrating an allelic contribution to human deafness in patients heterozygotic for SLC26A4 mutations [19].

## Materials and Methods

### Immunohistochemisty

Cryosections (10 µm) were treated for antigen-retrieval as described in [20] and blocked in 1% BSA/0.5% Triton X-100 in 1×PBS buffer. The primary antibodies (ab) were diluted in 0.2% BSA/0.1% Triton X-100 and incubated over night at 4°C. Three 10-minute washes in 1×PBS were followed by one hour incubation of diluted secondary ab at room temperature. Nuclei were visualized using ToPro3 1∶1000 (Molecular Probes Inc.). After washes in 1×PBS, the slides were mounted in ProLong antifade (Molecular Probes Inc.) and imaged with a Zeiss LSM 510 META confocal microscope. Tissues from at least three different animals were analyzed and the results were consistent. Antibodies were diluted as follows: goat anti-Foxi1 1∶500 (AbCam); rabbit anti-B1 subunit of H^+^-ATPase 1∶100; rabbit polyclonal antiserum against the a4-subunit of H^+^-ATPase, (Genosys); rabbit polyclonal anti E-subunit 1∶50 and rabbit polyclonal anti A1-subunit 1∶500 (supplied by Dr. S. Breton); donkey anti-goat Alexa488 1∶100 ( Molecular Probes Inc.); donkey anti-goat Cy3 1∶100 (Jackson ImmunoResearch Laboratories) and donkey anti-rabbit Rhodamine Red 1∶100 (Jackson ImmunoResearch Laboratories).

Combined cRNA in situ hybridization and immunofluorescence was performed essentially as described previously [12]. In brief, digoxigenin (DIG) labeled cRNA probes for A1 (corresponding to nt 233–550, GenBank Acc No. NM_007508) or E2 (corresponding to nt 338–886, GenBank Acc No NM_007510) was hybridized to mouse kidney cryosections overnight at 60°C and alkaline phosphatase conjugated sheep-anti DIG Fab fragments (Roche) were used for detection of bound probe. Upon completion of in situ hybridization procedure sections were treated for antigen retrieval using either incubation in 1% SDS in PBS at room temperature for 5 min or by boiling in a pressure cooker for 10 min in Tris-EDTA buffer (40 mM Tris, 0.1 mM EDTA, pH 8). Immunofluorescence was then performed using a rabbit anti-CAII antibody (1∶2000) and the nuclear marker Topro3 (Invitrogen). Stained sections were photographed on a Zeiss LSM 510 Meta system.

### Cell Culture, Transient Transfections and Luciferase Assays

The African green monkey kidney cell line COS-7 (American Type Culture Collection, USA) was cultured in DMEM, containing 4.5 g/liter glucose, 10% heat-inactivated calf serum, 100 U/ml Penicillin, 100 µg/ml Streptomycin (Invitrogen) and maintained in a humidified incubator with 5% CO_2_ at 37°C. Cells were grown in 24-well plates to approximately 50–80% confluence before transfection. Transient transfections were performed with FUGENE 6 transfection Reagent (Roche), using 120 ng of luciferase reporter plasmid co-transfected with 40, 80 and 100 ng expression vector, in accordance to manufacturer's instructions. Differences in transfection efficiencies were assayed by co-transfecting each well with 1.0 ng pRL-SV40 Renilla luciferase plasmid (Promega). After 48 hours of transfection cells were washed with cold PBS buffer, harvested with passive lysis buffer (Promega) and analyzed using a Dual-Luciferase Reporter Assay System (Promega) according to manufacturer's protocol. Luciferase activity was determined as fold induction relative to cells transfected with an expression vector void of insert, normalized to Renilla activity. Experiments were performed in triplicates.

### Plasmid Constructs and Mutants

*Expression construct:* The full-length human FOXI1 cloned into pcDNA3.1/GS expression vector (GeneStorm Clone ID) was obtained from ResGen, Invitrogen Corporation.

*Cloning of the 5ATP6V0A4 reporter construct:* 850 bp (corresponding to nt. 138109234–138110074, GenBank Acc no: NT_000007) of genomic sequence immediately upstream of exon 4 (ATG) of the ATP6V0A4 gene (GeneID: 50617) was PCR amplified from human DNA using custom ordered primers from Sigma-Genosys Online Ordering. Unique KpnI and HindIII restriction sites were incorporated at the 5′ and 3′ ends of the sequence, respectively, to simplify directed cloning into KpnI and HindIII sites in the reporter vector pGL3-basic (Promega). Primers: 1) FW: 5′ GTTGATAGGATGGTGAGTGTG 3′, 2) REV: 5′ CCAGTGAATCCGCAGGT 3′.

Based on information from GenBank this promoter region contains at least four putative FOXI1 binding sites, three in the region −561/−547 and one in −358/−352 relative to transcription start site. Introduction of the triple and double mutations (TTT>GGG and TT>GG) into the putative FOXI1 binding sites of the 5′ATP6V0A4 promoter was generated by PCR, using the Quick Change mutagenesis kit (Stratagene) according to the manufacturers protocol, with the following primers (mutations are underlined): MutFK1 - FW: 5′ AGA TAT ATA TAG GGA TTT ATT TAT TTT TGA GAT GGA GTC TGG C. MutFK1-REV: 5′ GCC AGA CTC CAT CTC AAA AAT AAA TAA ATC CCT ATA TAT ATC T. MutFK2 - FW: 5′ GAT ATA TAT ATT TAG GGA TTT ATT TTT GAG ATG GAG TCT GG. MutFK2-REV: 5′ CCA GAC TCC ATC TCA AAA ATA AAT CCC TAA ATA TAT ATA TCT. MutFK3 - FW: 5′ ATA TAT TTA TTT AGG GAT TTT TGA GAT GGA GTC TGG CTC T. MutFK3 -REV: 5′ AGA GCC AGA CTC CAT CTC AAA AAT CCC TAA ATA AAT ATA T. MutFK4 - FW: 5′ CAG GAT TTC ACC ATG GGG GCC AGG CTA GTC TC. MutFK4-REV: 5′ GAG ACT AGC CTG GCC CCC ATG GTG AAA TCC TG. The sequence of all constructs was verified by using ABI PRISM 310 Genetic Analyzer (Applied Biosystems).

### Electrophoretic mobility shift assay (EMSA)

*In vitro transcription and translation*: *In vitro* transcription/translation was performed according to the manufacturer's protocol using TnT Quick Coupled Transcription/translation Systems (Promega) with [^35^S] methionine and 2 µg expression plasmid.

*Electrophoretic mobility shift assay*: Single stranded oligonucleotides were ordered custom made (Sigma) and annealed in annealing buffer over night. Double stranded oligonucleotides were labeled using Klenow polymerase and [α-^32^P]dCTP and isolated from unincorporated nucleotides by gel extraction. For each binding reaction; 50,000 cpm of labeled probe was incubated with 2 µl of *in vitro* t/t *FOXI1* protein and 500 ng of poly dI∶dC in a binding-buffer containing 5 mM HEPES, pH 7.9, 26% glycerol, 1.5 mM MgCl_2_, 0.2 mM EDTA, 0.5 mM dithiothreitol, and 0.5 mM PMSF with 100 mM KCl at room temperature for 15 min. Competition experiments were performed in the presence of 10 to 50-fold molar excess of unlabeled wt or mutated probes, respectively. Samples were run in 6% non-denaturing polyacrylamide gels at 200 V in Tris-glycine buffer (50 mM Tris, pH 8.5; 380 mM glycine; 2 mM EDTA) at 4°C. Subsequently, gels were dried and subjected to autoradiography.

### Chromatin Immunoprecipitation (ChIP) assays

3T3-L1 cells were transfected with a Foxi1-His and ATP6V0A4 promoter constructs using FUGENE 6 transfection Reagent (Roche). Chromatin immunoprecipitation was performed using EZ ChIP kit (Upstate) according to manufacture's instructions, with addition of a 1 h pre clear with Protein G Sepharose. Immunoprecipitation were carried out overnight at 4°C with an Anti-6X His tag antibody (Abcam). ChIP primers were as follows : Foxi1 Forward 5′-GGATGGTGAGTGTGATATTT-3′; Foxi1 Reverse 5′-CATTTGAGGTCAGGAGTTTG-3′; Random Forward 5′-CCCGGTACCTTATAAGTTTCA-3′; Reverse 5′-GTCCCTCCTGGAAGGACAAA-3′.

### Statistics

All values are given as mean±SEM. Student's t-test was used for statistical analysis. A p-value of less than 0.05 was considered to be significant.
